# Machine Learning-Based Mortality Prediction in Chronic Kidney Disease among Heart Failure Patients: Insights and Outcomes from the Jordanian Heart Failure Registry

**DOI:** 10.3390/medicina60050831

**Published:** 2024-05-19

**Authors:** Mahmoud Izraiq, Raed Alawaisheh, Rasheed Ibdah, Aya Dabbas, Yaman B. Ahmed, Abdel-Latif Mughrabi Sabbagh, Ahmad Zuraik, Muhannad Ababneh, Ahmad A. Toubasi, Basel Al-Bkoor, Hadi Abu-hantash

**Affiliations:** 1 Cardiology Section, Internal Medicine Department, Specialty Hospital, Amman 12344, Jordan; alawaisheh@yahoo.com (R.A.); dabbas.aya.ad@gmail.com (A.D.); abdellatif.mughrabi.sabbagh@gmail.com (A.-L.M.S.); baselemad11@icloud.com (B.A.-B.); 2 Cardiology Section, Internal Medicine Department, King Abdullah University Hospital, Irbid 22110, Jordan; rkibdah@just.edu.jo (R.I.); ybahmed180@med.just.edu.jo (Y.B.A.); mjababneh@just.edu.jo (M.A.); 3 Cardiology Section, Internal Medicine Department, Jordan University Hospital, Amman 11942, Jordan; ahmadzuraaik@gmail.com (A.Z.); tubasi_ahmad@yahoo.com (A.A.T.); 4 Department of Cardiology, Amman Surgical Hospital, Amman 11180, Jordan; drhadi57@hotmail.com

**Keywords:** machine learning, heart failure, chronic kidney disease, mortality prediction, random forest classifier

## Abstract

*Background and Objectives:* Heart failure (HF) is a prevalent and debilitating condition that imposes a significant burden on healthcare systems and adversely affects the quality of life of patients worldwide. Comorbidities such as chronic kidney disease (CKD), arterial hypertension, and diabetes mellitus (DM) are common among HF patients, as they share similar risk factors. This study aimed to identify the prognostic significance of multiple factors and their correlation with disease prognosis and outcomes in a Jordanian cohort. Materials *and Methods:* Data from the Jordanian Heart Failure Registry (JoHFR) were analyzed, encompassing medical records from acute and chronic HF patients attending public and private cardiology clinics and hospitals across Jordan. An online form was utilized for data collection, focusing on three kidney function tests, estimated glomerular filtration rate (eGFR), blood urea nitrogen (BUN), and creatinine levels, with the eGFR calculated using the Cockcroft–Gault formula. We also built six machine learning models to predict mortality in our cohort. *Results:* From the JoHFR, 2151 HF patients were included, with 644, 1799, and 1927 records analyzed for eGFR, BUN, and creatinine levels, respectively. Age negatively impacted all measures (*p* ≤ 0.001), while smokers surprisingly showed better results than non-smokers (*p* ≤ 0.001). Males had more normal eGFR levels compared to females (*p* = 0.002). Comorbidities such as hypertension, diabetes, arrhythmias, and implanted devices were inversely related to eGFR (all with *p*-values <0.05). Higher BUN levels were associated with chronic HF, dyslipidemia, and ASCVD (*p* ≤ 0.001). Higher creatinine levels were linked to hypertension, diabetes, dyslipidemia, arrhythmias, and previous HF history (all with *p*-values <0.05). Low eGFR levels were associated with increased mechanical ventilation needs (*p* = 0.049) and mortality (*p* ≤ 0.001), while BUN levels did not significantly affect these outcomes. Machine learning analysis employing the Random Forest Classifier revealed that length of hospital stay and creatinine >115 were the most significant predictors of mortality. The classifier achieved an accuracy of 90.02% with an AUC of 80.51%, indicating its efficacy in predictive modeling. *Conclusions:* This study reveals the intricate relationship among kidney function tests, comorbidities, and clinical outcomes in HF patients in Jordan, highlighting the importance of kidney function as a predictive tool. Integrating machine learning models into clinical practice may enhance the predictive accuracy of patient outcomes, thereby supporting a more personalized approach to managing HF and related kidney dysfunction. Further research is necessary to validate these findings and to develop innovative treatment strategies for the CKD population within the HF cohort.

## 1. Introduction

Among cardiovascular diseases, heart failure (HF) is a progressive condition and one of the leading causes of death globally. The prevalence of cardiac diseases continues to rise, with HF significantly affecting healthcare systems and the quality of life of millions worldwide [1,2,3]. HF patients face a high risk of comorbidities like chronic kidney disease (CKD), arterial hypertension, and diabetes mellitus (DM) due to shared risk factors such as older age, obesity, and tobacco use [4,5,6]. These comorbidities correlate with higher mortality risk, increased healthcare costs, and worse outcomes [4,7]. Most HF cases coexist with at least two chronic conditions, underscoring the need for comprehensive care [4,7].

The Kidney Disease Improving Global Outcomes (KDIGO) guidelines define CKD as a structural or functional kidney abnormality lasting more than three months [8]. CKD is characterized by an estimated glomerular filtration rate (eGFR) below 60 and/or chronic kidney damage. Advanced stages of CKD are linked to poor prognosis, progression to end-stage renal disease (ESRD), cardiovascular disease (CVD), and death [8,9]. Patients with CKD have a higher prevalence of all forms of CVD, including myocardial infarction (MI), peripheral artery disease (PAD), and cerebrovascular accidents (CVA) [8]. Serum creatinine levels and eGFR estimates are vital markers in managing HF patients [8,10]. Elevated creatinine levels (>0.3 mg/dL) are associated with increased hospital stay and mortality [11]. GFR depends on renal plasma flow and autoregulatory mechanisms like vasoconstriction of afferent and efferent arterioles, which maintain GFR despite cardiac output decline [10,12].

CKD often exacerbates HF and acts as a disease multiplier, increasing hospitalization and mortality risks [4,10]. Cardiorenal syndrome illustrates the bidirectional nature of heart and kidney disease, where impairment of one organ can lead to dysfunction in the other [4]. The ‘low flow theory’ proposes that renal hypoperfusion triggers baroreceptor activation, RAAS activation, and tubular/glomerular damage [7,12]. In the Atherosclerosis Risk in Communities study, stage ≥3 CKD was associated with a 1.9-fold higher risk of HF [4,13,14].

Machine learning (ML) models have proven effective in managing cardiac diseases like HF [15,16]. Random Forest Classifiers aggregate predictions from multiple decision trees to create a more accurate predictive model. By reducing overfitting through averaging, the random forest approach enhances generalization and minimizes noise. This study aims to correlate kidney function measures (creatinine, BUN, and GFR) with outcomes in HF patients in Jordan and predict mortality using advanced machine learning techniques.

## 2. Methods

### 2.1. Study Design and Setting

Data were used from the Jordanian Heart Failure Registry, which was made by gathering medical records of acute and chronic HF patients who reviewed public or private cardiology clinics and hospitals across Jordan. This registry method has been previously described. The characteristics of the JoHF registry and the protocol of our study were registered at clinicaltrials.gov (NCT04829591). Institutional Review Board (IRB) approval was taken from appropriate centers. Records were progressively followed up at 3, 6, 9, and 12 months to register new lab work and to see if new complications developed and, therefore, if they resulted in any change in the medication list.

### 2.2. Data Collection

An online form was used to collect the data; this form was filled out by healthcare professionals who had access to the systems. It contained 10 sections, which involved the personal medical history of the patient, the status of his/her heart failure, all labs/procedures conducted on the patient, outcomes of treatment such as the need for mechanical ventilation, duration of hospital stay, and, lastly, whether morbidities or mortalities occurred. 

In this study, we focused on studying the factors associated with the 3 different kidney function tests and how, therefore, these tests affect the outcomes. The first measure studied was the estimated glomerular filtration rate (eGFR), which was calculated using the Cockcroft GFR formula, which depended on age, weight, and creatinine level. The second test was the blood urea nitrogen (BUN), which is considered normal if the level is in-between 7–20 mg/dL and abnormally high when it is >20 mg/dL. The last measure studied was the creatinine level, which is normal when the level is ≤115 µmol/L and high when levels are >115 µmol/L. Acute HF was used when patients’ record was taken from the ER, whether it was the first presentation of HF or if it was an acute-on-chronic HF exacerbation. Mechanical ventilation was documented if it was required during patients’ stay in the hospital. Meanwhile, death was a 30-day mortality.

### 2.3. Statistical Analysis

The data were entered using Microsoft Office Excel 2019, then imported and analyzed using IBM SPSS v.25 software. The relationship between exposures and outcome was tested using the *t*-test and Chi-square for continuous and categorical variables, respectively. A *p*-value <0.050 was considered statistically significant. We employed multiple imputations in R (version 4.4.0) using the “mice” package to address missing data, generating five imputed datasets through Predictive Mean Matching (PMM). Post-imputation, the same statistical analyses were performed on each dataset, with results pooled to provide final estimates. This approach ensured robust handling of missing data and enhanced the reliability of our findings, thereby accounting for the uncertainty introduced by the imputation of missing data.

### 2.4. Machine Learning Analysis

This study utilized a machine learning-based approach for predicting ‘Death’. For handling missing values, median imputation was applied to numerical columns, while the most frequent value was used for categorical columns. All numerical data were standardized using a StandardScaler (version 0.24), ensuring comparability across features. Recursive Feature Elimination with Cross-Validation (RFECV) was utilized, employing a Random Forest Classifier as the estimator to identify the most relevant features for predicting the target variable. The dataset was divided into training, validation, and test sets, with 40% of the data reserved for the combined validation and test sets. To mitigate class imbalance, the training set underwent resampling using the SMOTEENN technique, which combines Synthetic Minority Over-sampling (SMOTE) with Edited Nearest Neighbors (ENN), improving the model’s performance on minority classes.

A grid search with fivefold cross-validation was performed on the resampled training data to find the optimal set of hyperparameters. The best parameters identified were then used to train the final models. Six different machine learning models were evaluated to determine the most effective in predicting the target variable: Random Forest Classifier (RFC), Logistic Regression (LR), Support Vector Machine (SVM), AdaBoost Classifier, K-Nearest Neighbors (KNN), and Gradient Boosting Classifier (GBC). The models were assessed using several metrics, including accuracy, precision, recall, F1 score, and the Area Under the ROC Curve (AUC). To assess the influence of each feature on the model’s predictions, we utilized Permutation Feature Importance. This method evaluates the importance of each feature by observing the effect on model accuracy when the feature’s values are randomly shuffled, thereby disrupting the association between the feature and the outcome. This approach provides a straightforward and intuitive means of understanding feature relevance in the context of the model’s predictive capabilities.

## 3. Results

### 3.1. Characteristics of the Included Patients

Based on Jordan’s heart failure registry (JoHFR), 2151 heart failure patients were included; out of them, 644 medical records were used to study eGFR, 1799 records were used to study BUN, and lastly, 1927 records were used to study creatinine levels. Across all tests, age had a negative effect (*p* ≤ 0.001); meanwhile, surprisingly, smokers had better results than nonsmokers (*p* ≤ 0.001).

### 3.2. The Association between Patients’ Characteristics and Kidney Function

eGFR was normal in 371 patients but low in 273, as shown in Table 1. The total female count was 241, 121 of which had low eGFR. Meanwhile, the total male count was 403, with 152 patients with low eGFR. Therefore, males had more normal eGFR levels than females (*p* = 0.002). Moreover, a positive medical history of hypertension, diabetes, arrythmias, and implanted devices all affected eGFR levels inversely (all with *p*-values < 0.05). Patients with a family history of ASCVD or premature deaths had lower eGFR results compared to their counterparts (all with *p*-values <0.05). BUN was normal in 437 patients and high in 1362 patients, as shown in Table 2. In total, 174 males and 263 females had normal BUN levels. However, 594 males and 768 females had high BUN. For patients with chronic HF, dyslipidemia, and a previous history of ASCVD, BUN results were significantly higher (*p* ≤ 0.001 for all). Family history of premature deaths and ASCVD affected BUN levels negatively (*p* ≤ 0.001 for both). As for creatinine levels, 1113 patients had normal levels while 814 patients had abnormally high levels, as shown in Table 3. The distribution between males and females was as follows: the total number of males was 808, and 345 had high Cr. Meanwhile, females were 1119, out of them 469 had high Cr. Co-morbidities such as hypertension, diabetes, dyslipidemia, arrhythmias, and prior history of HF all led to higher Cr levels (all with *p*-values <0.05). Moreover, chronic HF patients had a higher reading of Cr compared to acute HF (*p* = 0.004). A positive family history of ASCVD was associated with high Cr (*p* ≤ 0.001). Regarding outcomes, patients with low eGFR levels had higher rates of need of mechanical ventilation (*p* ≤ 0.001) and death (*p* ≤ 0.001). On the other hand, higher levels of BUN did not affect the need for mechanical ventilation or death. Moreover, the need for mechanical ventilation and death rates were both higher in patients with high Cr levels.

### 3.3. Machine Learning Model Performance

**Figure 1:**
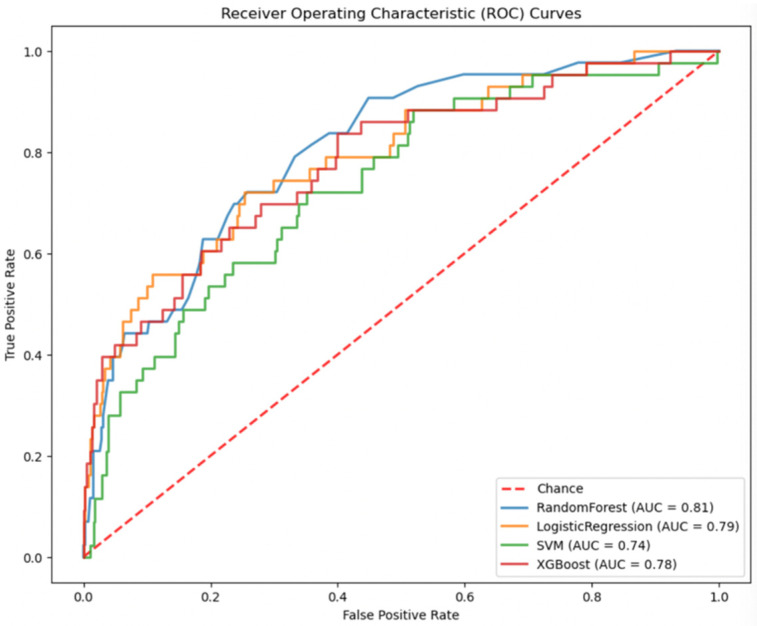
Receiver Operating Characteristic (ROC) Curves for the evaluated models. The dashed line represents the chance level of discrimination. Each model’s AUC is noted, with the Random Forest Classifier and Logistic Regression outperforming the SVM and XGBoost models marginally.

In the comparative analysis of predictive models shown in Table 4, the Random Forest Classifier (RFC) and eXtreme Gradient Boosting (XGBoost) showed similar accuracy (90.02%), with RFC exhibiting a slightly higher Area Under the ROC Curve (AUC) of 80.51%, compared to 78.21% for XGBoost (Figure 1). Logistic Regression (LR) demonstrated a balance between sensitivity (72.09%) and specificity (73.97%), with an AUC of 79.15%. The Support Vector Machine (SVM) model, while having lower accuracy (80.74%), showed a moderate AUC of 73.65%. The specificity of the models varied, with RFC showing the highest (96.39%) and LR the lowest (73.97%).

### 3.4. Feature Importance Analysis

**Figure 2:**
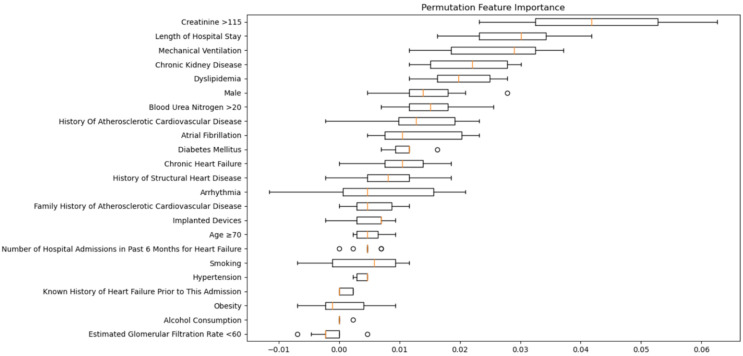
Permutation Feature Importance for the top-performing model, highlighting the mean decrease in model performance when each feature’s information is shuffled. The error bars represent the standard deviation of the permutation importance over multiple shuffles.

The Permutation Feature Importance analysis highlighted length of hospital stay and creatinine >115 as the most significant features influencing the model’s predictions (Figure 2). Other features, such as ‘Mechanical Ventilation,’ chronic kidney disease, and dyslipidemia, also showed a notable impact on the model’s performance. Conversely, factors like ‘Alcohol Consumption’ and ‘Estimated Glomerular Filtration Rate <60’ had the least impact, suggesting that their roles in predicting the target variable Death are less critical in the context of the models tested.

## 4. Discussion

The heart and kidneys play a significant role in maintaining fluid homeostasis and normal blood pressure in the body [7]. Individuals with CKD have mortality rates that are more than double the rate in the general population [8]. Our results show that patients with lower eGFR levels had higher mortality rates (19.1%), whereas HF patients with normal eGFR were 5.9%. Approximately more than 50% of patients with CKD suffer and die from CVD [7,8]. Cardio-renal syndrome is characterized based on which organ is primarily affected and whether it is acute or chronic damage [7]. In fact, patients with CKD are more likely to die from CVD than to progress to end-stage renal disease (ESRD) [8]. For that reason, improving or worsening the accuracy of GFR assessment has implications at the individual and population levels [7,17]. This study is about the multifaceted relationship between multiple kidney function measures, comorbidities, and clinical outcomes in heart failure patients, shedding light on several intriguing findings. For age, we observed a notable trend among older patients who exhibited higher Glomerular Filtration Rate (GFR) levels, increased Blood Urea Nitrogen (BUN) levels, and elevated creatinine (Cr) levels. Sex disparities in kidney health became apparent, with males displaying more normal eGFR levels than females, suggesting potential gender-related variations in kidney function. Females were more likely to have lower eGFR levels for a count of 121 out of 241; in comparison to males, only 152 out of 403 had low eGFR. This could be due to the higher body fat distribution in females; obesity, specifically central, plays a role in abnormal kidney function tests. Almost 30% of CKD Medicare patients have HF, compared with just 6% of Medicare patients without CKD [8]. Even the risk of incident HF is threefold greater in individuals with eGFR 90 mL/min/1.73 m^2^ [8,10]. This age–gender-related phenomenon raises questions about the intricate interplay between these factors and kidney health. The impact of common comorbidities was evident in our findings. Patients with diabetes mellitus displayed lower GFR and elevated Cr levels, indicating a potential link between diabetes and kidney dysfunction. Similarly, hypertensive patients exhibited lower GFR and higher Cr levels, underscoring the well-established association between high blood pressure and renal health. These results emphasize the need to proactively manage these conditions to preserve kidney function. Smokers surprisingly manifested lower BUN and Cr levels and higher GFR levels; 41.6% had normal eGFR on the other hand, and 19.5% had low eGFR. However, the unlimited adverse effects of smoking on overall health necessitate a cautious interpretation of these results. The smoker’s paradox is an interesting phenomenon where smokers sometimes appear to have better cardiovascular outcomes than non-smokers [18]. A few factors could explain this anomaly. For instance, smokers in this study may have been younger or had fewer baseline health risks compared to non-smokers, influencing kidney function measurements. Another possibility is survival bias; those with severe kidney disease or other health conditions might have quit smoking, leaving a relatively healthier group of current smokers. Confounding factors like socioeconomic status, diet, and healthcare access might also play a part. Moreover, chronic inflammation due to smoking could alter biochemical pathways, potentially masking kidney function problems. While the findings are intriguing, they need careful interpretation and more research to understand the relationship between smoking and kidney function fully. Patients with dyslipidemia had lower BUN (298 out of 437) and Cr levels, suggesting better kidney health. By contrast, atherosclerosis patients had lower GFR levels and higher bilirubin and Cr levels, highlighting the tangled connection between cardiovascular health and kidney function. Arrhythmias were notably more prevalent in patients with higher Cr levels and lower GFR levels.

For the clinical outcomes, represented by a number of hospital admissions, need for mechanical ventilation, and mortality rate, a prognostic value of kidney function in heart failure patients was evident. Elevated Cr levels were linked to a higher need for mechanical ventilation (6.7%) and an increased mortality rate (17%) vs. 3.4% and 5%, respectively. Similarly, lower GFR levels were associated with an increased need for mechanical ventilation and higher mortality, reinforcing the critical role of kidney function in predicting patient outcomes. CKD appears more common in HFpEF. Nevertheless, worse outcomes are mostly related to HFpEF and HFrEF [7]. The prevalence of CKD is higher among patients with acute decompensated heart failure (ADHF), ranging from 30 to 60%, depending on the definition used [10]. Natriuretic peptides can be elevated, among others, because of the low elimination of the molecules by the injured kidneys [7]. Increased levels of BNP and NT-proBNP may signal an elevated risk for accelerated progression of CKD to ESRD [7]. B-type natriuretic peptide (BNP) may be an additional marker to detect the involvement of kidneys in ventricular stress [7]. Remarkably, bilirubin levels did not significantly impact the number of admissions or the need for mechanical ventilation, signifying a distinct role for bilirubin in this context. A decrease in glomerular filtration rate (GFR) seems to be the most significant determinant of the overall progression of HF [7]. Damman et al. have reported that nearly half of the patients with heart failure suffer from decreased eGFR < 60 mL/min per 1.73 m^2^ were 18/1000 person years [7]. GFR estimation and creatinine concentration are nowadays recommended to assess and classify the impairment and dysfunction of the kidneys in HF [10]. In hospital, worsening renal function (WRF), which is often defined as an increase in creatinine of at least 0.3 mg/dL, is observed in 23% of HF patients [5,10]. The employment of machine learning models, particularly the Random Forest Classifier, has proven to be a powerful analytical tool in our study. The Random Forest’s higher AUC indicates its robustness in handling the complexity and nuances within our dataset, thus providing a reliable prediction for mortality in heart failure patients. This aligns with the evidence suggesting that machine learning models can capture interactions between clinical variables more effectively than traditional statistical methods. The model’s ability to discern the prognostic importance of variables like ‘Length of Hospital Stay’ and ‘Creatinine >115’ further emphasizes its utility in clinical settings. Moreover, the observed predictive power of the Random Forest Classifier underscores the potential for implementing such AI-driven tools in healthcare systems to aid in early detection and intervention, ultimately improving patient outcomes. However, it is critical to approach the integration of such models with caution, given their dependency on the quality and breadth of the data they are trained on, as well as their interpretability in clinical decision making. However, some limitations exist in the usage of creatinine serum level, as its levels are biased due to many factors, for example, age, gender, diet, and body mass index [10]. A common condition seen in advanced heart failure is muscle wasting, which shows a decrease in creatinine levels (with overestimation of GFR) [10]. However, to overcome their limits, several emerging glomerular and tubular biomarkers have been proposed over the last years, alongside imaging techniques that could complement the laboratory data exploring different pathophysiological pathways [10]. Both conventional HF risk factors and kidney-specific risk factors, such as anemia, acid/base imbalances, uremic toxins, bone mineral abnormalities, malnourishment, and myocardial stunning, are more common in patients with chronic kidney disease (CKD) [8]. Optimizing care for patients with HF and CKD hence requires a multidisciplinary therapeutic approach [4]. Clinical practice recommendations advocate the examination and treatment of iron-deficient anemia in this patient population since anemia is frequent in CKD and can independently increase the risk of hospitalization and mortality in persons with HF [4]. Early diagnosis and predictable prognosis help to systematically manage and discover new treatment methods [19]. Since conventional HF medications are underutilized in this population, more focused therapies may improve outcomes for individuals with renal disease [8]. It is important to acknowledge several limitations in this study. First, due to its observational design, this study could only establish associations rather than causation. Second, these associations may be influenced by various confounding variables. Third, this study’s limited follow-up period for assessing patient outcomes serves as an additional constraint.

## 5. Conclusions

In conclusion, this study underscores the complex interplay between kidney function measures, various comorbidities, and clinical outcomes in heart failure patients in Jordan. These findings emphasize the critical role of kidney functions as a prognostic indicator and stress the need for a comprehensive, multidisciplinary approach to managing both cardiac and renal aspects of heart failure. Machine learning models, particularly the Random Forest Classifier, have provided substantial predictive insights, highlighting its potential as a clinical decision-support tool. The model’s notable accuracy and AUC reflect its capability to navigate multifaceted clinical data and effectively identify key prognostic variables. This analytical prowess points towards a future where machine learning can assist clinicians in tailoring interventions more precisely for the heart failure population with coexisting CKD, thus fostering the advancement of personalized patient care. Nevertheless, the path forward mandates further research to refine these models, enhance their interpretability, and validate their efficacy across a wider spectrum of patient populations. Continued efforts are vital to evolve and substantiate innovative heart failure treatments for individuals with chronic kidney disease, ultimately aiming to improve outcomes and quality of life. Despite the absence of baseline data for hemoglobin and nutritional evaluation scores in this study, the current analysis provides valuable insights into the relationships between kidney function measures and patient outcomes. Future research should include these scores to refine predictive models and offer a comprehensive understanding of anemia management and nutritional status in CKD and HF populations.

## Figures and Tables

**Table 1:** The association between GFR and patient characteristics.

Variable, *n* (%)		Normal GFR mL/min/1.73 m^2^ (≥60) (*n* = 373 **)	Low GFR mL/min/1.73 m^2^ (<60) (*n* = 277 **)	*p*-Value
Gender	Male	251 (67.7)	152 (55.7)	0.002 *
	Female	120 (32.3)	121 (44.3)	
Age (years)	<40	22 (6.3)	3 (1.2)	<0.001 *
	40–49	30 (8.5)	2 (0.8)	
	50–59	76 (21.7)	28 (11.2)	
	60–69	106 (30.2)	72 (28.9)	
	≥70	117 (33.3)	144 (57.8)	
Hypertension	Yes	263 (77.1)	234 (89.3)	<0.001 *
	No	78 (22.9)	28 (10.7)	
Diabetes	No	118 (34.6)	61 (23.3)	0.003 *
	Yes	223 (65.4)	201 (76.7)	
Smoking	No	199 (58.4)	211 (80.5)	<0.001 *
	Yes	142 (41.6)	51 (19.5)	
Alcohol	No	334 (97.9)	259 (98.9)	0.387
	Yes	7 (2.1)	3 (1.1)	
Type of Heart Failure	Chronic	276 (75.2)	201 (73.1)	0.544
	Acute	91 (24.8)	74 (26.9)	
Dyslipidemia	No	121 (35.5)	97 (37.0)	0.697
	Yes	220 (34.5)	165 (63.0)	
Obesity	No	303 (88.9)	235 (89.7)	0.742
	Yes	38 (11.1)	27 (10.3)	
Family History of Premature Death	No	306 (89.7)	220 (84.0)	0.035 *
	Yes	35 (10.3)	42 (16.0)	
Family History of ASCVD	No	326 (95.6)	156 (59.5)	<0.001 *
	Yes	15 (4.4)	106 (40.5)	
History of ASCVD	No	47 (16.3)	40 (17.2)	0.783
	Yes	242 (83.7)	193 (82.8)	
History of Arrhythmias	No	261 (76.1)	157 (61.4)	<0.001 *
	Yes	109 (23.9)	118 (38.6)	
History of Implanted Device	No	283 (97.9)	220 (94.4)	0.034 *
	Yes	6 (2.1)	13 (5.6)	
History of Structural Heart Disease	No	271 (93.8)	213 (91.4)	0.303
	Yes	18 (6.2)	20 (8.6)	
History of HF	No	106 (28.6)	65 (23.7)	0.162
	Yes	264 (71.4)	209 (76.3)	
Admissions in Past 6 Months	0	206 (57.4)	141 (51.6)	0.474
	1	67 (18.7)	53 (19.4)	
	2	22 (6.1)	15 (5.5)	
	>2	64 (17.8)	64 (23.4)	
Mechanical Ventilation	No	255 (97.3)	207 (93.7)	0.049 *
	Yes	7 (2.7)	14 (6.3)	
Death	No	351 (94.1)	224 (80.9)	<0.001 *
	Yes	22 (5.9)	53 (19.1)	

Abbreviations: GFR: Glomerular Filtration Rate; HF: Heart Failure; ASCVD: Atherosclerotic Cardiovascular Disease. Values in parentheses indicate the percentage of patients within that subgroup relative to the total group size for that variable. * Statistical significance was determined with a *p*-value ≤ 0.05. ** Totals for some variables may not amount to the total group size due to missing values.

**Table 2:** The association between BUN and patient characteristics.

Variable, *n* (%)		Normal BUN mg/dL (*n* = 437)	High BUN mg/dL (>20) (*n* = 1362)	*p*-Value
Gender	Male	174 (39.8)	594 (43.6)	0.163
	Female	263 (60.2)	768 (56.4)	
Age (years)	<40	27 (6.3)	38 (3.0)	<0.001 *
	40–49	45 (10.5)	72 (5.8)	
	50–59	83 (19.3)	178 (14.3)	
	60–69	116 (27.0)	326 (26.1)	
	≥70	158(36.8)	633 (50.8)	
Hypertension	No	86 (20.6)	244 (19.0)	0.459
	Yes	331 (79.4)	1042 (81.0)	
Diabetes	No	135 (32.4)	379 (29.4)	0.243
	Yes	282 (67.6)	912 (70.6)	
Smoking	No	248 (59.5)	921 (71.6)	<0.001 *
	Yes	169 (40.5)	365 (28.4)	
Alcohol	No	412 (98.8)	1281 (99.6)	0.060
	Yes	5 (1.2)	5 (0.4)	
Type of HF	Chronic	330 (75.3)	896 (66.4)	<0.001 *
	Acute	108 (24.7)	454 (33.6)	
Dyslipidemia	No	119 (28.5)	627 (48.8)	<0.001 *
	Yes	298 (71.5)	659 (51.2)	
Obesity	No	378 (90.6)	1192 (92.7)	0.177
	Yes	39 (9.4)	94 (7.3)	
Family History of Premature Death	No	365 (87.5)	1259 (97.9)	<0.001 *
	Yes	52 (12.5)	27 (2.1)	
Family History of ASCVD	No	373 (89.4)	903 (70.5)	<0.001 *
	Yes	44 (10.6)	380 (29.0)	
History of ASCVD	No	47 (13.5)	218 (22.2)	<0.001 *
	Yes	302 (86.5)	766 (77.8)	
History of Arrhythmias	No	267 (65.3)	798 (67.4)	0.329
	Yes	170 (34.7)	567 (32.6)	
History of Implanted Device	No	334 (95.7)	946 (96.1)	0.720
	Yes	15 (4.3)	38 (3.9)	
History of Structural Heart Disease	No	329 (94.3)	920 (39.5)	0.609
	Yes	20 (5.7)	64 (6.5)	
History of HF	No	69 (15.8)	265 (19.3)	0.098
	Yes	368 (84.2)	1107 (80.7)	
Admissions in Past 6 Months	0	166 (39.1)	545 (41.1)	0.031 *
	1	90 (21.2)	241 (18.2)	
	2	43 (10.1)	93 (7.0)	
	>2	126 (29.6)	446 (33.6)	
Mechanical Ventilation	No	227 (93.8)	912 (95.3)	0.340
	Yes	15 (6.2)	45 (4.7)	
Death	No	392 (89.3)	1243 (90.0)	0.667
	Yes	47 (10.7)	138 (10.0)	

Abbreviations: BUN: Blood Urea Nitrogen; HF: Heart Failure; ASCVD: Atherosclerotic Cardiovascular Disease. Values in parentheses indicate the percentage of patients within that subgroup relative to the total group size for that variable. * Statistical significance was determined with a *p*-value ≤ 0.05.

**Table 3:** The association between creatinine level and patient characteristics.

Variable, *n* (%)		Normal Cr µmol/L (*n* = 1113)	High Cr µmol/L (>115) (*n* = 814)	*p*-Value
Gender	Male	463 (41.6)	345 (42.4)	0.730
	Female	650 (58.4)	469 (57.6)	
Age	<40	59 (5.8)	11 (1.4)	<0.001 *
	40–49	95 (9.3)	32 (4.2)	
	50–59	197 (19.3)	87 (11.3)	
	60–69	271 (36.5)	194 (25.3)	
	≥70	401 (39.2)	443 (57.8)	
Hypertension	No	42 (23.7)	111 (13.9)	<0.001 *
	Yes	779 (76.3)	688 (86.1)	
Diabetes	No	380 (37.1)	175 (21.9)	<0.001 *
	Yes	645 (62.9)	625 (78.1)	
Smoking	No	645 (63.2)	611 (76.5)	<0.001 *
	Yes	376 (36.8)	188 (23.5)	
Alcohol	No	1012 (99.1)	797 (99.7)	0.060
	Yes	9 (0.9)	2 (0.3)	
Type of HF	Chronic	792 (72.1)	538 (65.9)	0.004 *
	Acute	307 (27.9)	278 (34.1)	
Dyslipidemia	No	417 (40.8)	371 (46.4)	0.017 *
	Yes	604 (59.2)	428 (53.6)	
Obesity	No	935 (91.6)	738 (92.4)	0.540
	Yes	86 (8.4)	61 (7.6)	
Family History of Premature Death	No	976 (95.6)	753 (94.2)	0.190
	Yes	45 (4.4)	46 (5.8)	
Family History of ASCVD	No	979 (95.9)	401 (50.2)	<0.001 *
	Yes	42 (4.1)	398 (49.8)	
History of ASCVD	No	164 (20.1)	118 (18.8)	0.519
	Yes	650 (79.9)	510 (81.2)	
History of Arrhythmias	No	695 (62.7)	465 (56.6)	0.007 *
	Yes	414 (37.3)	357 (43.3)	
History of Implanted Device	No	781 (95.9)	605 (96.3)	0.703
	Yes	33 (4.1)	23 (3.7)	
History of Structural Heart Disease	No	761 (93.5)	587 (93.5)	0.989
	Yes	53 (6.5)	41 (6.5)	
History of HF	No	227 (20.5)	131 (15.9)	0.011 *
	Yes	882 (79.5)	692 (84.1)	
Admissions in Past 6 Months	0	457 (42.8)	324 (40.0)	0.368
	1	195 (18.2)	159 (19.7)	
	2	84 (7.9)	62 (7.7)	
	>2	333 (31.1)	264 (32.6)	
Mechanical Ventilation	No	648 (96.6)	559 (93.3)	0.008 *
	Yes	23 (3.4)	40 (6.7)	
Death	No	1064 (95.0)	688 (83.0)	<0.001 *
	Yes	56 (5.0)	141 (17.0)	

Abbreviations: Cr: creatinine; HF: heart failure; ASCVD: atherosclerotic cardiovascular disease. Values in parentheses indicate the percentage of patients within that subgroup relative to the total group size for that variable. * Statistical significance was determined with a *p*-value ≤ 0.05.

**Table 4:** Performance metrics of the used algorithms for death prediction.

Model	Accuracy	AUC	Sensitivity	Specificity
Random Forest Classifier	90.02%	80.51%	32.56%	96.39%
Logistic Regression	73.78%	79.15%	72.09%	73.97%
Support Vector Machine	80.74%	73.65%	46.51%	84.54%
eXtreme Gradient Boosting	90.02%	78.21%	39.53%	95.62%

## Data Availability

The datasets used and/or analyzed during this current study are available from the corresponding author on reasonable request.

## References

[1] Yoo B.S. (2014). Clinical Significance of B-type Natriuretic Peptide in Heart Failure. J. Lifestyle Med..

[2] Bhagat A.A., Greene S.J., Vaduganathan M., Fonarow G.C., Butler J. (2019). Initiation, Continuation, Switching, and Withdrawal of Heart Failure Medical Therapies During Hospitalization. JACC. Heart Fail..

[3] Beezer J., Al Hatrushi M., Husband A., Kurdi A., Forsyth P. (2022). Polypharmacy definition and prevalence in heart failure: A systematic review. Heart Fail. Rev..

[4] Vijay K., Neuen B.L., Lerma E.V. (2022). Heart Failure in Patients with Diabetes and Chronic Kidney Disease: Challenges and Opportunities. Cardiorenal Med..

[5] Polonsky T.S., Bakris G.L. (2019). Heart Failure and Changes in Kidney Function: Focus on Understanding, Not Reacting. Heart Fail. Clin..

[6] GBD 2017 Disease and Injury Incidence and Prevalence Collaborators (2018). Global, regional, and national incidence, prevalence, and years lived with disability for 354 diseases and injuries for 195 countries and territories, 1990-2017: A systematic analysis for the Global Burden of Disease Study 2017. Lancet.

[7] Szlagor M., Dybiec J., Młynarska E., Rysz J., Franczyk B. (2023). Chronic Kidney Disease as a Comorbidity in Heart Failure. Int. J. Mol. Sci..

[8] Tuegel C., Bansal N. (2017). Heart failure in patients with kidney disease. Heart.

[9] Matsushita K., Ballew S.H., Wang A.Y., Kalyesubula R., Schaeffner E., Agarwal R. (2022). Epidemiology and risk of cardiovascular disease in populations with chronic kidney disease. Nat. Rev. Nephrol..

[10] Grande D., Gioia M.I., Terlizzese P., Iacoviello M. (2018). Heart Failure and Kidney Disease. Adv. Exp. Med. Biol..

[11] Ronco C., Haapio M., House A.A., Anavekar N., Bellomo R. (2008). Cardiorenal syndrome. J. Am. Coll. Cardiol..

[12] Schefold J.C., Filippatos G., Hasenfuss G., Anker S.D., von Haehling S. (2016). Heart failure and kidney dysfunction: Epidemiology, mechanisms and management. Nature reviews. Nephrology.

[13] Ronco C., Bellasi A., Di Lullo L. (2018). Cardiorenal Syndrome: An Overview. Adv. Chronic Kidney Dis..

[14] Roehm B., McAdams M., Hedayati S.S. (2022). Novel Biomarkers of Kidney Disease in Advanced Heart Failure: Beyond GFR and Proteinuria. Curr. Heart Fail. Rep..

[15] Nowak C., Ärnlöv J. (2020). Kidney Disease Biomarkers Improve Heart Failure Risk Prediction in the General Population. Circ. Heart Fail..

[16] Tomasoni D., Adamo M., Lombardi C.M., Metra M. (2019). Highlights in heart failure. ESC Heart Fail..

[17] Levey A.S., Titan S.M., Powe N.R., Coresh J., Inker L.A. (2020). Kidney Disease, Race, and GFR Estimation. Clin. J. Am. Soc. Nephrol. CJASN.

[18] Fonarow G.C., Abraham W.T., Albert N.M., Stough W.G., Gheorghiade M., Greenberg B.H., O’Connor C.M., Nunez E., Yancy C.W., Young J.B. (2008). A smoker’s paradox in patients hospitalized for heart failure: Findings from OPTIMIZE-HF. Eur. Heart J..

[19] Han X., Zhang S., Chen Z., Adhikari B.K., Zhang Y., Zhang J., Sun J., Wang Y. (2020). Cardiac biomarkers of heart failure in chronic kidney disease. Clin. Chim. Acta; Int. J. Clin. Chem..

